# Bias due to Preanalytical Dilution of Rodent Serum for Biochemical Analysis on the Siemens Dimension Xpand Plus

**DOI:** 10.3389/fvets.2018.00003

**Published:** 2018-02-15

**Authors:** Jennifer L. Johns, Kaitlin A. Moorhead, Jing Hu, Roberta C. Moorhead

**Affiliations:** ^1^Department of Biomedical Sciences, College of Veterinary Medicine, Oregon State University, Corvallis, OR, United States; ^2^Department of Comparative Medicine, Stanford School of Medicine, Stanford, CA, United States

**Keywords:** clinical pathology, laboratory animal science, methodological bias, dilution effect, serum biochemistry

## Abstract

Clinical pathology testing of rodents is often challenging due to insufficient sample volume. One solution in clinical veterinary and exploratory research environments is dilution of samples prior to analysis. However, published information on the impact of preanalytical sample dilution on rodent biochemical data is incomplete. The objective of this study was to evaluate the effects of preanalytical sample dilution on biochemical analysis of mouse and rat serum samples utilizing the Siemens Dimension Xpand Plus. Rats were obtained from end of study research projects. Mice were obtained from sentinel testing programs. For both, whole blood was collected *via* terminal cardiocentesis into empty tubes and serum was harvested. Biochemical parameters were measured on fresh and thawed frozen samples run straight and at dilution factors 2–10. Dilutions were performed manually, utilizing either ultrapure water or enzyme diluent per manufacturer recommendations. All diluted samples were generated directly from the undiluted sample. Preanalytical dilution caused clinically unacceptable bias in most analytes at dilution factors four and above. Dilution-induced bias in total calcium, creatinine, total bilirubin, and uric acid was considered unacceptable with any degree of dilution, based on the more conservative of two definitions of acceptability. Dilution often caused electrolyte values to fall below assay range precluding evaluation of bias. Dilution-induced bias occurred in most biochemical parameters to varying degrees and may render dilution unacceptable in the exploratory research and clinical veterinary environments. Additionally, differences between results obtained at different dilution factors may confound statistical comparisons in research settings. Comparison of data obtained at a single dilution factor is highly recommended.

## Introduction

Biochemical analysis of patient or research animal samples is frequently limited by inadequate sample volume. This problem of “short-volume” samples is particularly common with samples from rodents, due to their small size and low absolute blood volume. Survival (non-terminal or interim) blood sampling of laboratory rodents, when possible, is preferred over terminal blood sampling to reduce animal numbers in accordance with “3R’s” philosophy (1–3). However, lower sample volumes are available from survival blood samples versus terminal blood samples, compounding the problem of short-volume samples from rodents. Blood samples obtained *via* terminal procedures generally have higher available volume but are not repeatable, i.e., resampling is not possible in the case of a volume-limited sample.

One potential solution to the problem of short-volume samples is preanalytical sample dilution (“predilution”). Dilution of serum samples is commonly performed either manually or automatically when analyte values are above assay range (exceeding the upper limit of detection or quantification) on initial biochemical analysis. In contrast, when samples are prediluted prior to analysis, the analyte values are not known ahead of time, and dilution may cause analyte results to fall below the lower limit of detection. Dilution-induced overall error can also occur due to imprecision introduced by manual pipetting error, bias due to sample matrix effects, alterations in enzyme activity, and other factors (4). Published information on the potential impact of sample predilution on biochemical analytes in rodents is limited. The use of predilution of rodent samples in regulatory research is generally discouraged (4). However, exploratory research and clinical veterinary diagnostics have different requirements and purposes for testing that may allow for the use of predilution to maximize sample volume and reduce animal use. Laboratories may be currently performing sample predilution out of necessity, with little or no data on potential dilution-induced error in measurement.

In this prospective study, we evaluate the effects of predilution on biochemical analysis of mouse and rat serum samples on the Siemens Dimension Xpand Plus, and summarize trends in dilution-induced bias through a range of dilution factors.

## Materials and Methods

### Sample Selection and Handling

Rats were obtained from various ends of study research projects. A total of 38 adult (>12 weeks of age) rats scheduled for service euthanasia by the investigators were obtained from Stanford University vivaria and transferred to an animal use protocol approved by the Stanford University Institutional Animal Care and Use Committee and overseen by the Veterinary Service Center for this and related purposes. Genetic background and medical history of all rats was unknown, and male and female animals were equally represented. Mouse serum samples were previously banked from animals used in the disease surveillance program. All mice were CD-1 strain females. Mice and rats were euthanized by CO_2_ asphyxiation following the 2013 AVMA Guidelines on Euthanasia (5). All blood was collected by cardiocentesis following euthanasia into empty collection tubes and allowed to clot at room temperature for 30 min. Serum was harvested following centrifugation of coagulated blood. Rat serum samples were refrigerated at 4°C until use within 24 h of blood collection. Mouse samples were frozen at −30°C for a maximum of 1 year prior to use. 5–6 mouse serum samples were thawed and pooled to use as a single “pooled sample”; a total of 26 pooled samples were used. The Stanford vivaria are accredited by the Association for Assessment and Accreditation of Laboratory Animal Care International. Husbandry was performed in accordance with the Guide for the Care and Use of Laboratory Animals (6) and the Public Health Service Policy on Humane Care and Use of Laboratory Animals (7).

### Predilution and Biochemical Analysis

Manual predilution of mouse and rat serum samples was performed in the following manner. Each dilution level was prepared separately from the undiluted sample to avoid any magnifying of pipetting error *via* serial dilutions. Dilution levels were prepared from “×2” dilution (1:1 ratio of serum to diluent, indicated as dilution factor 2) through “×10” dilution (1:9 ratio of serum to diluent, indicated as dilution factor 10). Serum was mixed well *via* pipetting prior to preparing each dilution level. Per instrument manufacturer specifications, ultrapure water was used as diluent for all analytes [albumin (ALB), urea nitrogen (BUN), total calcium (CA), cholesterol (CHOL), creatinine (CREA), electrolytes (potassium, sodium and chloride), glucose (GLU), high-density lipoproteins (HDL), magnesium, phosphorus, total bilirubin (TBI), total CO_2_ (TCO_2_), total protein (TP), triglycerides (TGL), uric acid (URCA)] except enzymes. Siemens enzyme diluent (Siemens Healthcare Diagnostics, Inc., Tarrytown, NY, USA) was used to dilute samples for alanine aminotransferase (ALT), alkaline phosphatase (ALP), amylase (AMY), aspartate aminotransferate (AST), creatine kinase (CK), gamma-glutamyl transferase (GGT), lactate dehydrogenase (LDH), and lipase (LIP) measurement. One diluted sample was prepared for each original sample and diluent (e.g., one sample to analyze all enzymes was diluted to a factor of two by mixing serum 1:1 with enzyme diluent; one sample to analyze all other analytes was diluted to a factor of two by mixing serum 1:1 with water). Manual pipetting was performed by a single operator using Rainin (Mettler-Toledo, LLC; Columbus, OH, USA) and Gilson (Gilson, Inc., Middleton, WI, USA) pipettes that are calibrated annually per manufacturer recommendations. Additional evaluation of dilution-induced effects was performed by diluting QC reagents (Bio-Rad MultiQual Liquid Unassayed reagent; Bio-Rad, Hercules, CA, USA) to dilution factors 2–10. Level 3 QC reagent was diluted as above in water or enzyme reagent for measurement of all analytes included in the reagent. Additional evaluation of dilution-induced effects specifically in calcium measurement was performed by diluting two QC reagent levels, level 1 and level 1.5 (consisting of a 1:1 mixture of levels 1 and 2) in water prior to analysis. All biochemical analyses were performed on the Siemens Dimension Xpand Plus with software version 10.1.2 (Siemens Healthcare Diagnostics, Inc.). Specific methodology information for each biochemical test is shown in Table 1. Low-volume or pediatric software settings were used whenever available for specific analytes and electrolyte dilutions were tested using both serum and urine analysis settings.

**Table 1:** Methods used in automated serum biochemical analysis on the Siemens Dimension Xpand.

Analyte	Method
Albumin (g/dL)	Bromcresol green dye binding; colorimetric detection

ALP (IU/L)	Enzymatic assay detecting transphosphorylation activity of ALP on *p*-nitrophenylphosphate; bichromatic rate detection

ALT (IU/L)	Two-step enzymatic assay detecting transamination activity of ALT on l-alanine and subsequent oxidation of NADH; bichromatic rate detection

Amylase (IU/L)	Enzymatic assay detecting activity of AMY on chromogenic substrate linked with maltotriose bichromatic rate detection

AST (IU/L)	Two-step enzymatic assay detecting transamination activity of AST on l-aspartate and subsequent oxidation of NADH; bichromatic rate detection

BUN (mg/dL)	Two-step enzymatic assay detecting urea *via* urease generation of ammonia and subsequent oxidation of NADH; bichromatic rate detection

Calcium (mg/dL)	Colorimetric assay detecting calcium complexed with *o*-cresolphthalein complexone; bichromatic endpoint detection

Chloride (mmol/L)	Ion selective electrode (contained in integrated multisensor)

Cholesterol (mg/dL)	CHOL esterase hydrolysis, generation of hydrogen peroxide by oxidation of free CHOL; polychromatic endpoint technique

Creatine kinase (IU/L)	Coupled enzymatic assay detecting CK transphosphorylation activity and subsequent reduction of NADP; bichromatic rate detection

Creatinine (mg/dL)	Modified kinetic Jaffe reaction utilizing picrate chromophoric substrate in presence of strong base; bichromatic rate detection

GGT (IU/L)	Enzymatic assay detecting glutamyl transferase activity of GGT on γ-glutamyl-3-carboxy-4-nitranilide; bichromatic rate detection

Glucose (mg/dL)	Modified hexokinase method; GLU is phosphorylated then glucose-6-phosphate is oxidized and NAD reduced; bichromatic endpoint detection

HDL (mg/dL)	Dextran complexing step, enzymatic detection of hydrogen peroxide generated by oxidation of HDL-C by CHOL esterase and CHOL oxidase; bichromatic endpoint detection

LDH (IU/L)	Enzymatic assay detecting LDH activity on l-lactate by reduction of NAD; bichromatic rate detection

Lipase (IU/L)	Enzymatic assay detecting LIP hydrolysis of ester substrate and subsequent generation of chromogenic free methylresorufin; bichromatic rate detection

Phosphorus (mg/dL)	Detection of phosphate complexed to molybdate, subsequent reduction of complex by *p*-methylaminophenol sulfate and bisulfite; bichromatic endpoint detection

Potassium (mmol/L)	Ion selective electrode (contained in integrated multisensor)

Sodium (mmol/L)	Ion selective electrode (contained in integrated multisensor)

Total bilirubin	Modified diazo method detecting diazo-bilirubin *via* reaction of solubilized (conjugated and delta form) bilirubin with diazotized sulfanilic acid (unconjugated bilirubin is first solubilized); bichromatic endpoint detection

Total carbon dioxide (mmol/L)	Two-step enzymatic assay detecting bicarbonate anion *via* reaction with phosphoenolpyruvate and subsequent oxidation of NADH analog; bichromatic detection

Total protein (g/dL)	Modified biuret method utilizing cupric ion complexed with peptide linkages in basic solution with tartrate as complexing agent; bichromatic endpoint detection

Triglycerides (mg/dL)	Enzymatic assay utilizing lipoprotein LIP generation of glycerol, subsequent phosphorylation of glycerol and oxidation of glycerol-3-phosphate to generate hydrogen peroxide followed by peroxidase generation of quinoneimine; bichromatic endpoint detection

Uric Acid (URCA) (mg/dL)	Enzymatic assay detecting URCA conversion to allantoin by uricase; bichromatic endpoint detection

### Statistical Analysis

Data were initially exported into Microsoft Excel 2013 (Microsoft Office Professional Plus, Microsoft Corp., Redmond, WA, USA). Total systematic error (i.e., bias), was calculated as [(value (diluted sample))/(value (undiluted sample))] − 1 and expressed as a percentage (8). Mean, 2SD and 95% CI values were calculated for bias data and charts displaying this analyzed data across dilution factors for each analyte were constructed. Data were evaluated for manual pipetting error across all analytes at a single dilution level in a single diluent for a single sample (e.g., marked error present in all enzyme values for a single sample at dilution factor 2). If such error was greater than 150% over the mean error for all relevant analytes at that dilution factor, the data for that sample were excluded. Individual analyte results were generally included in the statistical analysis if reported, except for data not reported by the analyzer or data points with instrument flags indicating an analysis error. Subsequent analysis was performed in GraphPad Prism 6 for Windows (GraphPad Software Inc., La Jolla, CA, USA). Linear regression was performed across dilution factors for individual analytes to calculate slope and *y*-intercept, assess 95% CI around slope and *y*-intercept, and determine if either it was significantly non-zero *via F*-test and/or 95% CI evaluation. Slopes and *y*-intercepts of data pairs were compared *via* linear regression. When slopes differed markedly, differences between *y*-intercepts could not be quantified.

## Results

Outlier exclusion identified three samples for which all data at a single dilution factor in a single diluted sample showed clear pipetting error, and the data were discarded. Two of the pooled mouse serum samples could not be evaluated past dilution factor 5 for some analytes due to volume limitations. Numerous data points fell below assay range particularly at higher dilution factors for some analytes; in all cases, these assay range errors precluded reporting of data. Data on GGT measurement were not included in the statistical analysis of serum dilution as most GGT values in undiluted rodent serum samples were 0, precluding any useful evaluation of dilution effect. Data on electrolyte measurement were not included in the statistical analysis or figures as values were often not reported by the analyzer in analysis of either diluted serum or diluted QC reagent, and an “assay range” error was instead obtained. This error occurred when both “serum” or “urine” analyzer software settings were used, and occurred starting at low dilution factors. Precision studies using diluted QC reagent confirmed that the “assay range” error occurs under the “serum” analysis setting starting at dilution factor 2 to 3 (data not shown). As such, we do not recommend analysis of electrolytes in diluted serum samples at any dilution factor.

**Figure 1:**
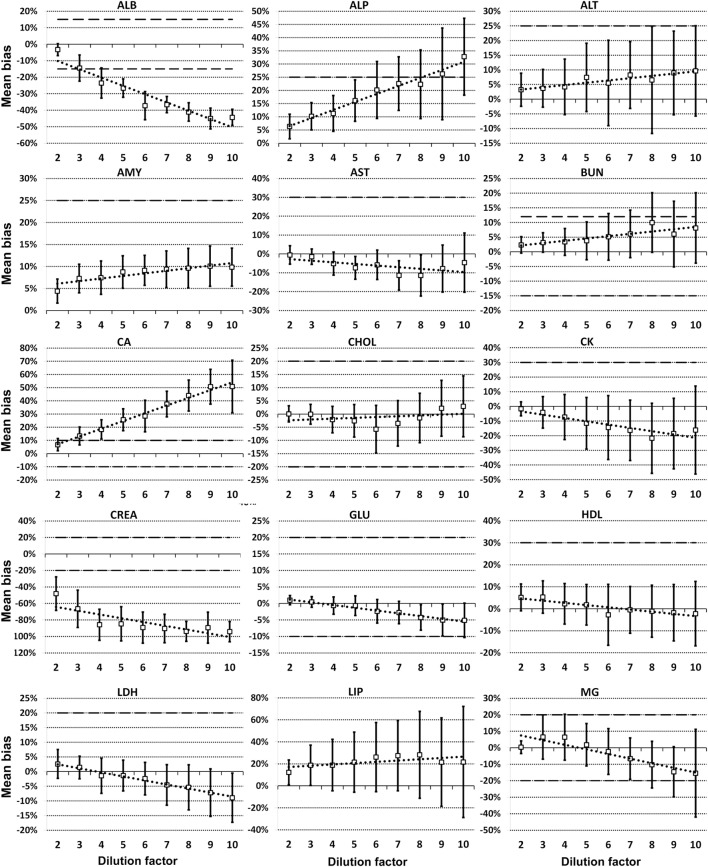
Mean bias data points (open boxes) and 95% CI (solid bars) around the mean at dilution factors 2−10 for analytes listed. Dashed lines indicate limits of clinically acceptable error for specific analytes. Linear regression lines (dotted lines) are graphed for each analyte.

**Figure 2:**
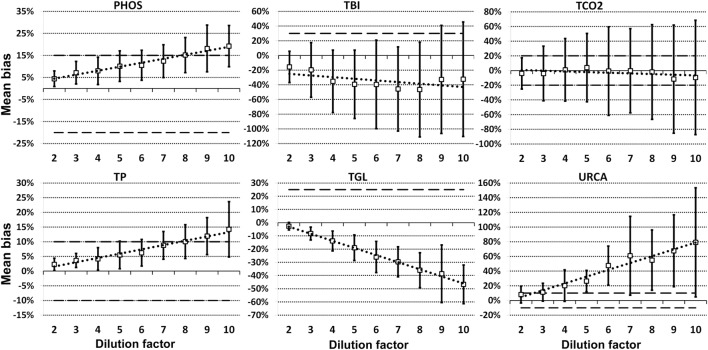
Mean bias data points (open boxes) and 95% CI (solid bars) around the mean at dilution factors 2−10 for analytes listed. Dashed lines indicate limits of clinically acceptable error for specific analytes. Linear regression lines (dotted lines) are graphed for each analyte.

For each analyte at each dilution factor mean bias, 2SD of mean bias and 95% CI around the mean bias were calculated (Table 2). The highest dilution factor at which (a) mean bias or (b) 2SD of mean bias remained within recommended clinically allowable limits were determined for each analyte (Table 3). All clinically allowable total error limits were obtained from published ASVCP guidelines (3), with the exception of total error limits for HDL obtained from Westgard QC’s website on CLIA Requirements for Analytical Quality (9). Allowable limits of total error were not available for LIP. Although mean bias was often acceptable at higher dilution factors, most analytes were unacceptably altered at lower dilution factors based on 2SD of mean bias exceeding clinically established limits. In addition, several analytes (e.g., TBI and TGL) had substantial dilution-induced negative bias that, although not surpassing clinically acceptable limits, might impact measurement and/or interpretation even at low dilution factors. Figures 1 and 2 display mean values with 95% CI around mean, along with linear regression lines and clinically allowable limits of total error, for each analyte at each dilution factor.

**Table 2:** Analysis of (a) mean bias, (b) 2SD of mean bias, and (c) 95% CI around mean bias, for analytes at dilution factors 2 through 10.

	Dilution factor								
Analyte	2	3	4	5	6	7	8	9	10
**(a) Mean bias (%)**									
ALB	−3.3	−14.5	−23.5	−26.6	−37.3	−36.6	−41.1	−45.0	−44.3
ALP	6.3	10.2	11.3	16.1	20.2	22.6	22.3	26.3	32.8
ALT	3.2	−19.0	−8.5	7.4	5.5	8.2	6.6	9.0	9.6
AMY	4.4	7.3	7.4	8.7	9.1	9.4	9.6	10.1	9.8
AST	−0.5	−1.5	−5.2	−7.4	−5.8	−11.4	−11.5	−7.8	−4.6
BUN	2.4	3.2	3.4	3.8	5.1	6.1	10.0	6.0	8.1
CA	6.8	13.3	18.3	25.7	28.5	37.5	44.0	50.7	50.9
CHOL	0.1	−0.1	−2.1	−2.6	−5.7	−3.5	−1.5	2.2	2.9
CK	−1.7	−4.1	−7.3	−11.5	−14.4	−16.3	−21.7	−18.6	−16.1
CREA	−48.1	−66.5	−85.9	−84.7	−89.3	−90.4	−93.9	−89.4	−94.2
GLU	1.0	0.4	−0.6	−0.7	−2.4	−2.8	−4.2	−5.0	−5.1
HDL	5.1	5.3	2.2	1.8	−2.8	−0.5	−1.1	−1.8	−2.2
LDH	2.6	1.4	−1.4	−1.4	−2.4	−4.6	−5.4	−7.2	−8.9
LIP	27.2	45.2	55.6	64.5	71.8	75.0	85.4	84.9	97.4
MG	0.3	6.5	6.4	1.8	−2.3	−6.6	−10.3	−14.6	−15.4
PHOS	4.4	7.2	8.0	10.1	10.5	12.4	15.2	18.1	19.2
TCO_2_	−4.0	−4.1	0.9	4.0	−0.7	−0.2	−2.0	−11.7	−9.4
TGL	−2.7	−8.3	−13.9	−19.0	−26.0	−29.6	−36.0	−38.7	−46.7
TBI	−15.6	−19.7	−35.3	−39.3	−39.5	−45.7	−46.5	−32.7	−32.4
TP	2.3	3.6	4.2	5.5	6.2	8.8	10.0	11.9	14.2
URCA	8.1	11.3	20.2	26.3	47.5	60.9	54.9	67.7	79.1

**(b) 2SD of mean bias (%)**									
ALB	14.4	31.0	35.9	21.9	32.6	18.8	20.9	22.7	18.3
ALP	18.9	20.9	27.5	31.6	41.9	39.4	49.2	64.5	56.1
ALT	22.5	76.5	58.8	46.3	56.6	43.8	69.0	53.0	59.3
AMY	10.9	13.0	15.0	14.5	13.2	16.0	17.1	17.5	16.8
AST	19.8	16.6	24.6	24.2	30.6	30.6	41.7	47.2	61.1
BUN	11.3	13.1	18.1	25.2	30.9	31.3	38.5	40.9	45.4
CA	18.6	26.4	24.9	28.4	39.9	32.5	39.3	44.1	67.5
CHOL	12.2	14.6	19.9	24.2	35.1	33.2	35.5	38.5	43.7
CK	15.2	34.2	49.0	55.4	68.4	63.9	74.1	74.7	93.2
CREA	80.7	87.7	64.7	71.7	63.8	58.5	40.9	63.5	42.4
GLU	5.5	6.3	10.3	11.6	13.8	13.0	14.6	17.5	19.6
HDL	20.4	24.5	30.4	30.5	45.8	35.1	39.1	42.4	48.5
LDH	17.0	13.3	20.6	18.2	19.0	23.7	26.3	27.6	29.0
LIP	38.4	62.6	80.1	93.4	106.4	107.9	133.6	136.1	170.7
MG	12.7	44.4	42.6	40.5	43.0	38.9	43.2	43.8	79.1
PHOS	14.1	19.8	24.0	27.0	26.0	28.5	29.9	38.2	35.1
TCO_2_	18.0	29.1	35.9	40.7	43.1	45.7	52.8	21.3	75.9
TGL	11.3	19.2	29.6	37.9	45.8	42.9	49.9	79.3	55.3
TBI	85.4	144.6	148.9	162.6	204.3	194.1	218.1	249.1	269.7
TP	8.3	9.4	14.9	18.4	17.4	18.2	21.6	22.9	35.6
URCA	29.7	31.6	54.7	37.1	67.9	137.0	100.4	111.9	177.8

**(c) 95% CI of mean bias (%)**									
ALB	3.6	7.9	9.2	5.6	8.5	4.9	5.6	6.3	4.9
ALP	4.7	5.2	6.8	7.9	10.8	10.1	13.0	17.4	14.6
ALT	5.6	19.5	16.8	11.6	14.6	11.4	18.2	14.3	15.4
AMY	2.7	3.3	3.8	3.7	3.4	4.2	4.5	4.6	4.3
AST	4.9	4.1	6.1	6.0	7.8	7.8	10.9	12.5	15.7
BUN	2.8	3.3	4.6	6.4	8.0	8.1	10.2	11.2	12.0
CA	4.6	6.8	7.3	8.3	11.9	9.7	11.8	13.2	19.9
CHOL	3.0	3.7	5.0	6.2	9.0	8.6	9.4	10.6	11.5
CK	4.8	10.7	15.4	17.6	21.8	20.6	23.9	24.1	30.0
CREA	20.2	22.6	18.9	20.7	18.9	17.3	12.1	18.8	12.2
GLU	1.4	1.6	2.6	3.0	3.6	3.4	3.8	4.8	5.2
HDL	6.1	7.3	9.2	9.2	13.9	10.6	11.8	12.8	14.7
LDH	4.9	3.9	6.0	5.3	5.6	6.9	7.7	8.1	8.4
LIP	11.2	18.3	23.4	27.3	31.4	31.9	39.5	40.2	50.4
MG	3.8	13.4	13.9	12.9	13.9	12.5	14.1	15.2	26.6
PHOS	3.5	5.1	6.2	6.9	6.8	7.5	8.0	10.6	9.3
TCO_2_	7.4	13.1	23.4	28.2	34.5	36.6	42.3	17.1	52.6
TGL	2.8	4.9	7.5	9.7	11.8	11.3	13.2	21.8	14.6
TBI	21.3	37.2	42.6	46.5	60.4	57.3	64.4	73.6	77.9
TP	2.1	2.4	3.8	4.7	4.5	4.7	5.8	6.3	9.4
URCA	11.4	12.1	21.4	14.5	26.6	53.7	41.0	49.0	74.3

**Table 3:** Highest dilution factor at which (a) mean bias; (b) 2SD of mean bias is within clinically acceptable limits as defined in the text.

Analyte	(a) Highest dilution factor: mean bias within clinically acceptable limit	(b) Highest dilution factor: 2SD of mean bias within clinically acceptable limit
ALB	3	2
ALP	8	3
ALT	10	2
AMY	10	10
AST	10	5
BUN	10	2
CA	2	None
CHOL	10	4
CK	10	2
CREA	None	None
GLU	10	10
HDL	10	3
LDH	10	3
MG	10	2
PHOS	7	2
TCO_2_	10	2
TGL	10	2
TBI	3	None
TP	7	3
URCA	2	None

Evidence of proportionate bias due to increasing dilution factor was found in all except five analytes, based on line equation slopes differing significantly from zero when analyzed *via* linear regression (Table 4). Proportionate bias as indicated by line equation slopes *via* linear regression analysis was not consistently negative or positive, even within subsets of analytes (e.g., all enzymes). These data indicate that prediction of proportionate dilution-induced bias in one analyte based on dilution-induced bias in other analytes is not advisable. *y*-intercept values were significantly non-zero, consistent with constant bias due to overall dilution-induced alterations and/or bias present starting at dilution factor 2, in 12 of the 23 analytes evaluated (Table 4).

**Table 4:** Slope and *y*-intercept data from linear regression analysis of mean bias for each analyte at dilution factors 2 through 10.

Analyte	Slope ± SE	Slope significantly non-zero	*y*-Intercept ± SE	*y*-Intercept significantly non-zero
ALB	−5.019 ± 0.5952	**<0.0001**	−0.1358 ± 3.888	NS
ALP	3.041 ± 0.2034	**<0.0001**	0.4296 ± 1.329	NS
ALT	2.34 ± 1.013	NS	−11.59 ± 6.618	NS
AMY	0.5857 ± 0.1148	**0.0014**	4.918 ± 0.75	**Yes**
AST	−0.8665 ± 0.4134	NS	−0.981 ± 2.701	NS
BUN	0.7872 ± 0.1767	**0.003**	0.6159 ± 1.154	NS
CA	5.861 ± 0.2499	**<0.0001**	−4.54 ± 1.633	**Yes**
CHOL	0.3092 ± 0.3597	NS	−3.012 ± 2.35	NS
CK	−2.245 ± 0.3965	**0.0008**	1.038 ± 2.59	NS
CREA	−4.576 ± 1.216	**0.007**	−55.03 ± 7.943	**Yes**
GLU	−0.8368 ± 0.05015	**<0.0001**	2.861 ± 0.3276	**Yes**
HDL	−0.9993 ± 0.1987	**0.0015**	6.667 ± 1.298	**Yes**
LDH	−1.381 ± 0.07365	**<0.0001**	5.262 ± 0.481	**Yes**
LDL	3.315 ± 0.5248	**0.0004**	−30.78 ± 3.428	**Yes**
Lipase	7.832 ± 0.6578	**<0.0001**	20.45 ± 4.297	**Yes**
MG	−2.803 ± 0.4679	**0.0005**	13.02 ± 3.056	**Yes**
PHOS	1.814 ± 0.1046	**<0.0001**	0.8016 ± 0.6834	NS
TBI	−2.251 ± 1.191	NS	−20.57 ± 7.778	**Yes**
TCO_2_	−0.911 ± 0.5915	NS	2.462 ± 3.864	NS
TGL	−5.371 ± 0.1334	**<0.0001**	7.683 ± 0.8711	**Yes**
TP	1.463 ± 0.09231	**<0.0001**	−1.363 ± 0.603	NS
URCA	9.291 ± 0.7364	**<0.0001**	−13.97 ± 4.81	**Yes**

*A *p*-value less than 0.05 (values in bold) for slope not equal to 0 is considered statistically significant and consistent with proportionate bias due to increasing dilution factor. *y*-intercept is determined to be significantly non-zero (bold font) if 95% CI (not shown) does not include 0, consistent with constant bias due to dilution and/or bias even at lowest dilution factor. NS = not significant*.

Dilution-induced effects were also evaluated *via* serial dilution of Siemens QC reagent level 3 (Table S1 in Supplementary Material) to evaluate dilution effects at higher baseline analyte concentrations, a more optimal situation as diluted analytes are more likely to remain within the linear range for each assay, and to assess dilution effects in a different matrix environment. The data indicate that dilution-induced bias was repeatable in some analytes using QC reagent instead of serum. Regression analysis found that slopes and *y*-intercept values for dilution-induced bias were significantly altered due to use of QC reagent instead of serum. Higher starting analyte concentrations lessened but did not eliminate dilution-induced proportionate bias in several analytes that occur at low physiologic concentrations in serum (e.g., ALB, CA).

Dilution-induced bias in calcium measurement was further investigated *via* serial dilution of QC reagent at two starting calcium concentrations (QC level 1, CA = 5.8 mg/dL; QC level 1.5, CA = 7.7 mg/dL) that are substantially lower than the starting calcium concentration for QC level 3 (CA = 12.5 mg/dL). Dilution-induced positive proportionate bias in calcium measurement occurred with both reagents (Table 5), confirming that bias is at least partly independent of serum sample-related factors. Bias was marked at lower dilution factors than when QC level 3 was analyzed, consistent with more severe dilution-induced bias when sample calcium concentrations are initially lower.

**Table 5:** Dilution-induced bias in calcium measurement evaluated *via* serial dilution of Siemens QC reagent levels 1 and 1.5.

Bias (%)	Total calcium (CA)	CA

Dilution factor	QC level 1	QC level 1.5
2	6.90	1.30
3	15.52	5.19
4	39.66	19.48
5	32.76	27.27
6	44.83	19.48
7	63.79	36.36
8	56.90	38.96
9	70.69	49.35
10	86.21	51.95
Slope ± SE	9.138 ± 0.8803	6.386 ± 0.5997
95% CI of slope	7.056 to 11.22	4.967 to 7.804
*y*-Intercept ± SE	−8.464 ± 5.75	−10.61 ± 3.918
95% CI of *y*-intercept	−22.06 to 5.132	−19.87 to −1.345
Slope differs from slope for QC level 3	***p* < 0.0001**	***p* = 0.0026**
*y*-Intercept differs from y-intercept for QC level 3	ND	ND

*Regression lines are compared statistically to that obtained from serial dilution of Siemens QC reagent level 3. Significant differences in slope and *y*-intercept between data from QC reagent level 1 or 1.5 and QC reagent level 3 are determined by *p*-value less than 0.05 (values in bold). ND = not determined*.

## Discussion

This biochemistry predilution study found that dilution-induced error occurred in the large majority of analytes evaluated, based on boundaries of error exceeding clinically acceptable limits. Proportionate bias due to increasing dilution factor was common. The utility of data from prediluted samples depends heavily on intended use of the data. The stricter requirements of regulatory research environments generally preclude predilution of samples for purpose of extending sample volume (4). In contrast, the exploratory research environment often requires data from only a few analytes versus an entire panel, and data are generally used in statistical comparisons only. As such, cautious use of data from prediluted samples may be acceptable in an exploratory research or early drug discovery environment if validation data are “fit for purpose” (10), as we proposed in our prior study examining the effects of predilution of rodent blood samples on hematology analytes (11). In that study, several analytes had dilution-induced error that was within statistically acceptable limits; predilution may, therefore, be utilized for those analytes under strict conditions including predilution of all samples (including all experimental and control groups) to the same dilution factor and use of a single anticoagulant.

In this study, we used both statistical evaluation of the data and comparison to clinically acceptable limits for error in biochemistry analysis to assess acceptability of data from prediluted samples. The dilution factor limits shown in Table 3 include conclusions drawn from both mean bias and from 2SD around the mean; the latter value is more clinically useful as individual samples had dilution-induced error that often greatly exceeded the mean bias. Clinical decision-making based on a single sample can be markedly affected by inappropriate predilution, and conservative use of predilution is indicated. Individual laboratories will have to determine what degree of dilution-induced error is acceptable based on intended use of data. Additionally, this study only assessed rodent samples on a single chemistry analyzer/platform, and evaluation of other analyzers and specimens from other species is needed.

Little published work exists on the effects of predilution of animal blood samples for clinical pathology analysis. In an abstract report of the effects of threefold predilution on rat blood sample analysis, dilution-induced bias outside of published recommendations occurred in multiple biochemical analytes, including TBI, urea, CA, TP, ALB, CHOL, and GLU (12). Most of these analytes were also unacceptable at this dilution factor in our study. Interestingly, GLU was one of only two analytes with acceptable dilution-induced error, based on 2SD of mean error remaining within published limits, through a dilution factor of 10 in our study. A brief study of predilution of pooled plasma from healthy mice of a single strain found values from diluted samples to be “well within the ranges of the undiluted samples,” apart from electrolytes (13). Another abstract report found that predilution of mouse blood samples was generally acceptable for biochemical analysis at a 1:3 dilution level (14). Differences in results for biochemical evaluation of prediluted rodent samples may be partly attributable to differences in methods and operators (different analyzers and diluents used; different laboratory personnel).

The goal of the study was to evaluate predilution under the typical sample conditions of the exploratory research laboratory. Samples accordingly mirrored these conditions, with all rat samples taken from mixed populations including healthy, ill and experimentally altered animals of various strains and with various induced mutations. Mouse serum samples were obtained from generally healthy animals; to add variability, these serum samples were frozen prior to analysis for times ranging from weeks to up to 1 year. This approach was intended to partly mimic biologic variation in serum biochemistry values, as freezing has been shown to alter serum analyte values (15). These animals and sample were, therefore, more likely to have one or more biochemical analytes outside of reference intervals; values below reference intervals may negatively impact the acceptability of dilution-induced bias by lowering analyte concentrations below limits of detection. Use of only healthy rodents of a single strain for such analysis would likely improve the acceptability of dilution-induced bias as biochemical values are more likely to fall within reference intervals for these animals, and it is possible that SD and 95% CI values around mean error would be, therefore, reduced. QC materials were included in this study for two reasons: (1) to evaluate predilution in an “optimal” setting of high starting analyte concentrations and (2) to assess whether dilution-induced error was also present in reference materials that are subject to different matrix effects than patient samples. Our evaluation of dilution-induced error using QC reagents with high analyte concentrations ideally minimized contribution due to values falling at the low end of the linear range. These higher starting analyte concentrations reduced dilution-induced error in some analytes but error was still present when reference materials were utilized.

Outlier data were defined and removed conservatively, as this was not a reference interval study. In some cases, potential pipetting-induced error could not be conclusively differentiated from other error and all such data were retained in order to better represent conditions in a working diagnostic laboratory. Individual labs can assess error induced by manual pipetting for sample dilution; if multiple personnel are involved, inter-operator variability can also be evaluated. Manual pipetting error should be reduced when possible by appropriately training personnel and standardizing procedures and equipment. Precision was evaluated as a separate variable in a study of predilution effects on avian plasma, and increased imprecision with dilution was found for some analytes (16). Precision was not quantified separately in our study and is included as one component of overall dilution-induced error. Numerous data points were excluded for some analytes (e.g., CREA and TBI) due to assay range errors preventing the analyzer from reporting data. These assay range errors occurred most often at higher dilution factors, and were preceded by markedly increased bias in the same analyte at a lower dilution factor. As such, these data points are not expected to substantially alter the conclusions for acceptable bias, as the degree of bias was deemed unacceptable in each analyte at a lower dilution factor than that at which most assay range error occurred.

Lower-volume analyzers can offer a potential solution to short-volume sample issues in clinical pathology testing. Some smaller hematology analyzers offer useful lower-volume analysis options, but benchtop biochemistry analyzers often require similar or higher sample volumes than their full-size counterparts (4). Several analyzers include a predilution mode or low-volume settings, e.g., the Advia 1800 (Siemens Healthcare Diagnostics, Inc.) offers predilution but the overall volume required for a full panel is similar to that for other models without predilution. In this study, we used the lowest-volume settings (e.g., pediatric mode) when available. However, low-volume settings and/or predilution mode tend to minimally decrease the overall sample volume required, in part because the “dead space” requirement of the analyzer remains unchanged. The total volume required by the Siemens Dimension Xpand to run a full panel, including all analytes tested in this study plus a “dead space” of 35 µL, is 258 µL.

Dilution-induced error in biochemical analysis may partly stem from sample-related effects, e.g., matrix effects. Immunoassays are most commonly affected by matrix effects but clinical chemistry methodology can be affected as well, resulting in the need to harmonize clinical chemistry data *via* use of commutable samples (10, 17). Serum and plasma can both create matrix effects; these effects can be non-linear across dilution, requiring comparisons in immunoassay data to be made only between samples diluted to the same degree (18). As predicted, dilution-induced bias (particularly proportionate bias) in the level 3 QC reagent was decreased compared to bias in diluted serum samples. Interestingly, variability can increase when using “artificial” reference materials as compared to fresh or frozen patient samples, a phenomenon attributed to matrix effects in the reference materials (19, 20). Translation of dilution-induced error in QC reagent analysis into expected dilution-induced error in serum sample analysis is inadvisable.

In summary, dilution-induced error in biochemical analysis of rodent serum is variable and analyte-dependent. Dilution-induced error is considered unacceptable at low dilution factors for numerous analytes including electrolytes, calcium, ALB, CREA, TBI, and URCA. Dilution for biochemical analysis of some analytes might be utilized in the exploratory research environment under strict conditions, including evaluation of error for a laboratory’s specific instrumentation and laboratory personnel, and cautious use of the generated data.

## Author Contributions

JJ performed all data analysis and prepared the manuscript, tables, and figures. KM, JH, and RM performed all sample analyses and dilutions, compiled experimental data and data on instrument and assay validation, and contributed to the manuscript.

## Conflict of Interest Statement

The authors declare that the research was conducted in the absence of any commercial or financial relationships that could be construed as a potential conflict of interest.

## Abbreviations

ALB, albumin; ALT, alanine aminotransferase; ALP, alkaline phosphatase; AMY, amylase; AST, aspartate aminotransferate; BUN, urea nitrogen; CA, total calcium; CHOL, cholesterol; CK, creatine kinase; CREA, creatinine; GGT, gamma-glutamyl transferase; GLU, glucose; HDL, high-density lipoproteins; LDH, lactate dehydrogenase; LIP, lipase; MG, magnesium; PHOS, phosphorus; TBI, total bilirubin; TCO_2_, total CO_2_; TP, total protein; TGL, triglycerides; URCA, uric acid.
